# Supplementary material for: TDP-1 and FUST-1 co-inhibit exon inclusion and control fertility together with transcriptional regulation
Source: Nucleic Acids Res. 2023 Aug 17;51(18):9610–28. doi: 10.1093/nar/gkad665 (PMC10570059; doi:10.1093/nar/gkad665)
Supplement: gkad665_Supplemental_Files [file gkad665_supplemental_files.zip › supplementary_figures_revision_2.pdf]

SUPPLEMENTAL FIGURES

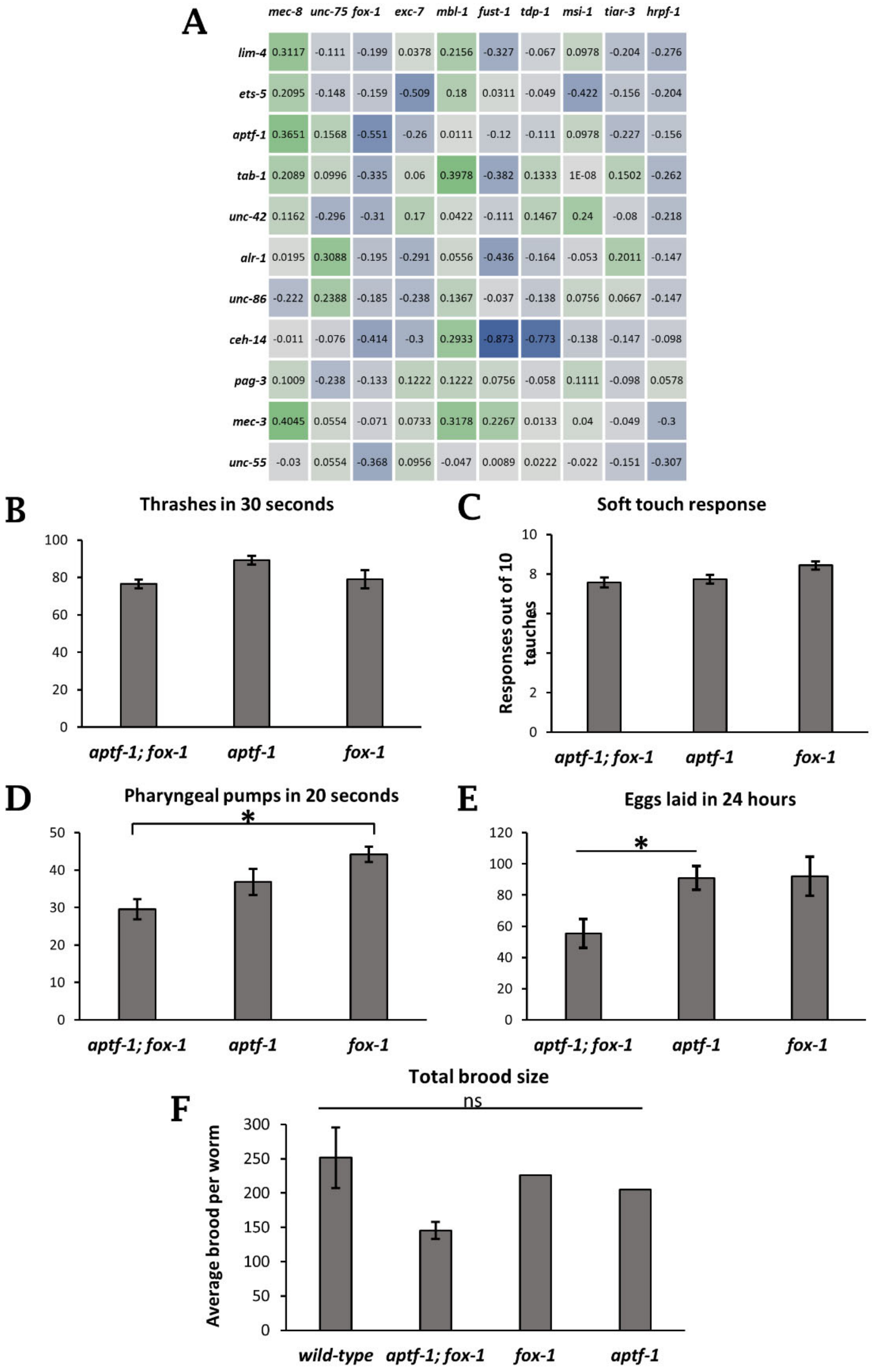

Supplemental figure 1. ***aptf-1; fox-1*** **double mutants exhibit slowed rates of egg laying and pharyngeal pumping compared to single mutants.** (A) Heatmap as shown in Fig 1F, but with no filtering out of |ε|<0.4 so that the full range of relative fitness values can be observed. (B) Number of thrashes per L4 worm in 30 seconds. No significant

differences were observed between groups. (C) Soft touch assays were performed with an eyelash hair pick. No significant differences were observed. (D) Number of pharyngeal pumps in 20 seconds was counted. Asterisk indicates a significant reduction in pumps in *aptf-1; fox-1* compared to *fox-1* single mutants, $p < 0.05$. (E) Early adult worms were individually plated and given 24 hours to lay eggs. Asterisk indicates significantly reduced eggs were counted on *aptf-1; fox-1* plates compared to *aptf-1,* $p < 0.05$. (F) Total brood size produced over the adult lifespan of each worm was quantified. No significant difference was observed between groups.

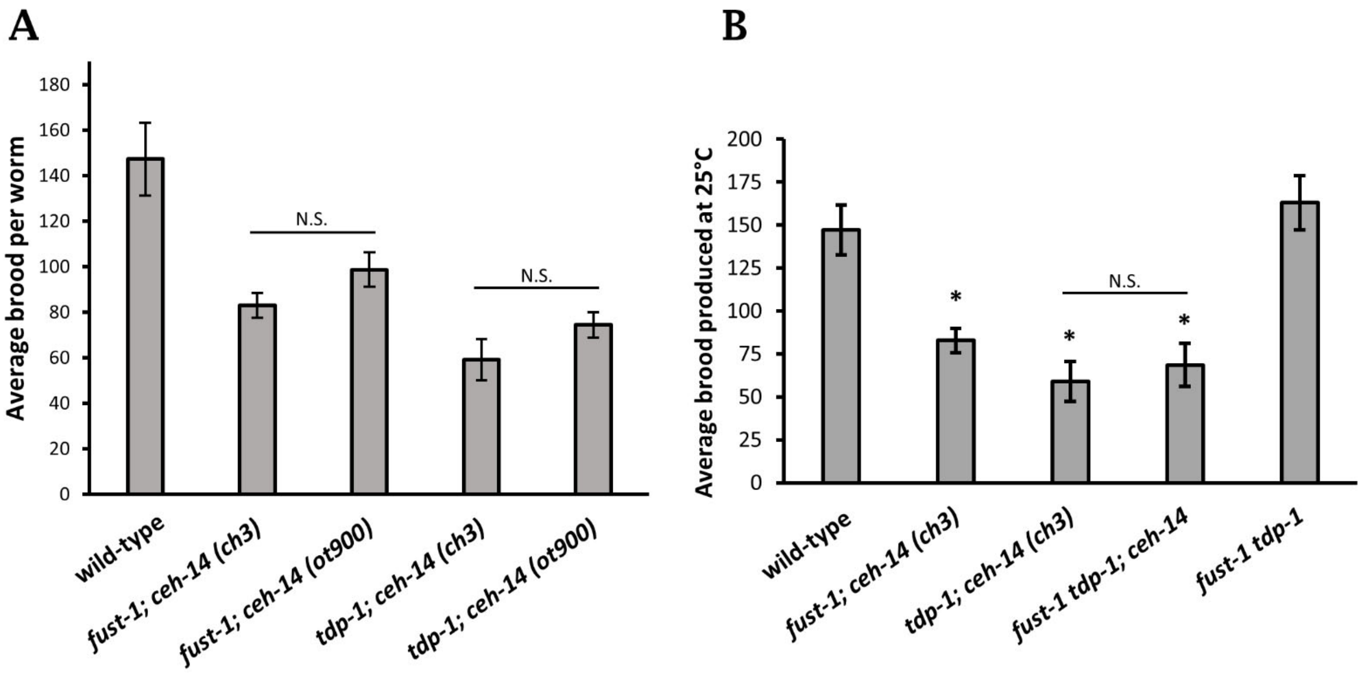

Supplemental Figure 2. *fust-1; ceh-14* and *tdp-1; ceh-14* phenotypes are recapitulated with alternative alleles. (A) Both double mutants were generated using *ceh-14 (ot900)*, which contains a full deletion of *ceh-14*. No significant differences in brood size were measured between double mutants generated with the original *ceh-14 (ch3)* allele and those generated with *ceh-14 (ot900)*. (B) A *fust-1 tdp-1; ceh-14* triple mutant did not exhibit a worsened brood size defect compared to *fust-1; ceh-14* and *tdp-1; ceh-14* double mutants. Asterisk indicates significant difference from wild-type, $p<0.05$. *fust-1 tdp-1; ceh-14* brood size is not significantly different from that of wild-type.

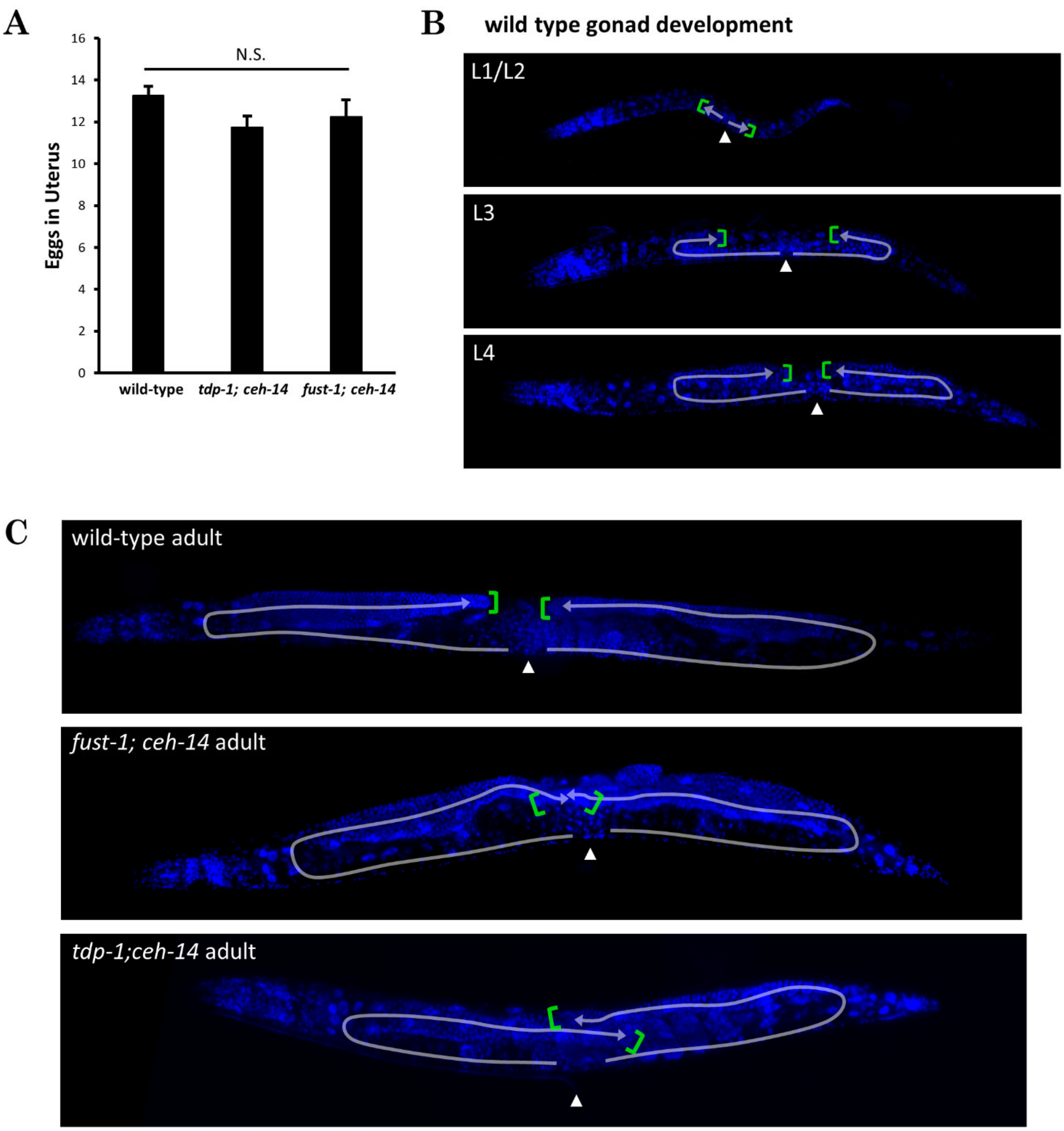

Supplemental Figure 3. *fust-1; ceh-14* and *tdp-1; ceh-14* exhibit defects in adult hermaphrodite gonad. (A) Double mutants do not exhibit egg retention. (B) Representative images of developing wild-type DAPI-stained larval hermaphrodites. (C) Representative images of DAPI-stained wild-type and double mutant adult hermaphrodites. *fust-1; ceh-14* and *tdp-1; ceh-14* exhibit overlapping distal tips. Brackets indicate path of gonad development. Arrow heads denote location at midbody where vulva is present in adult. Green brackets indicate approximate location of distal tip.

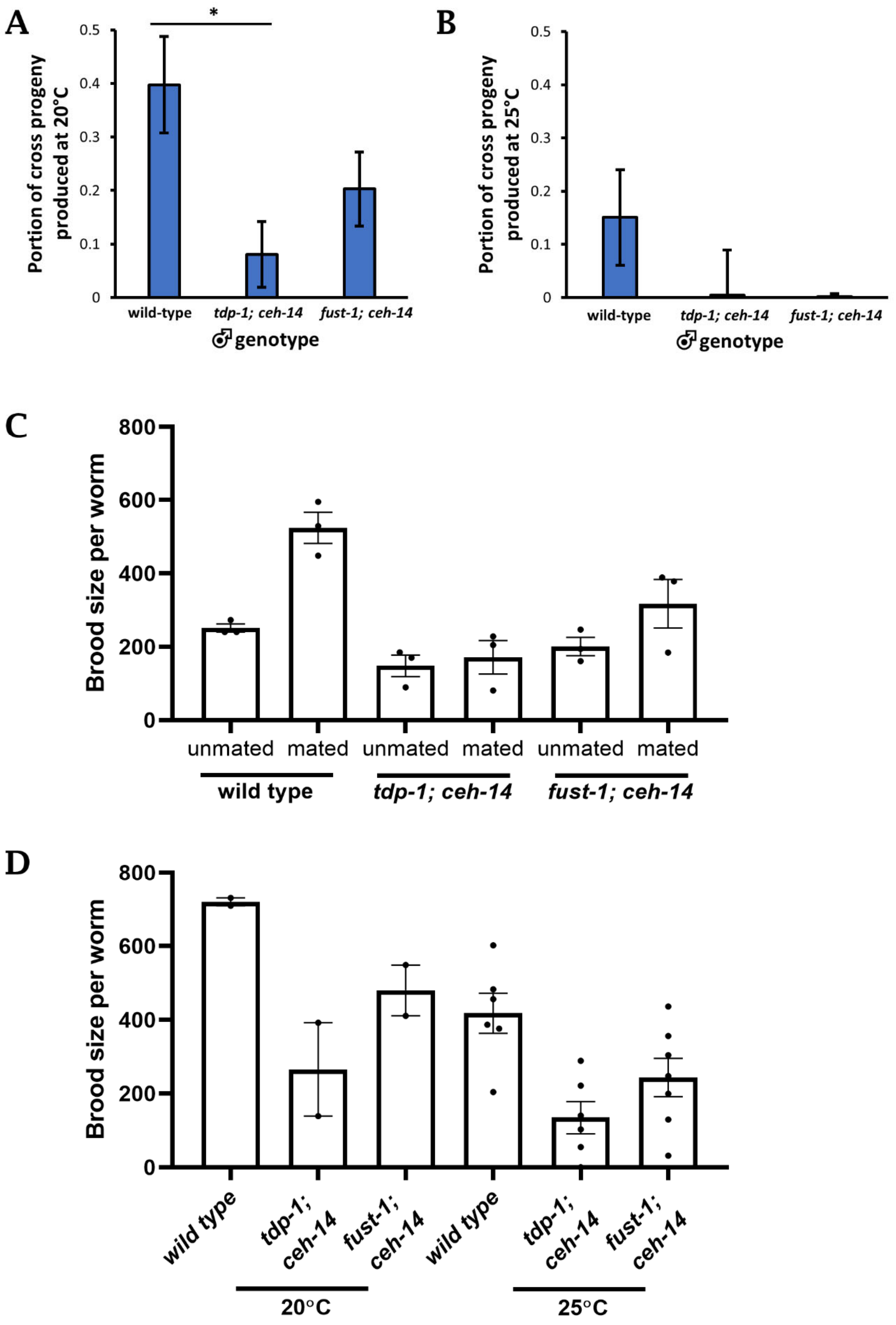

Supplemental Figure 4. Male mating defects in double mutants. *tdp-1; ceh-14* and *fust-1; ceh-14* males maintained at 20°C (A) and at 25°C (B) were paired with wild-type hermaphrodites, and production of cross-progeny was measured. Asterisk indicates significant difference from wild-type cross progeny, $p<0.05$. (C) Male

mating with *fog-2 (q71)* feminized germline mutants. Double mutant males produced smaller brood sizes than wild type. (D) Wild-type males were mated with either wild-type or double mutant hermaphrodites.

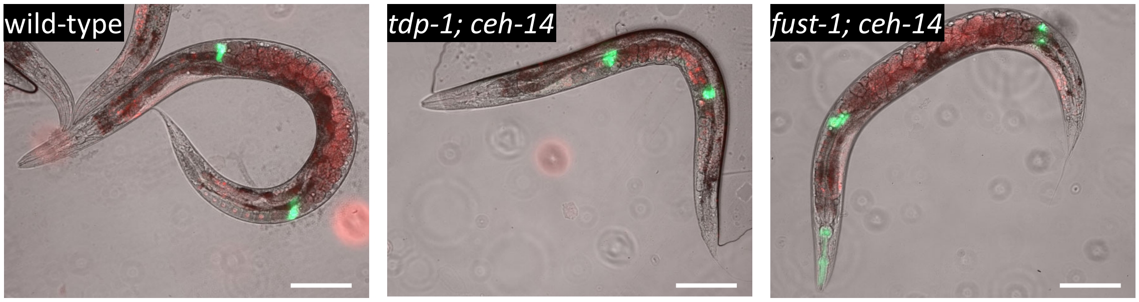

Supplemental Figure 5. Spermatheca of *tdp-1; ceh-14* and *fust-1; ceh-14* double mutants are morphologically similar to wild-type. UX993 worms containing spermatheca GFP and germline RFP were crossed into *tdp-1; ceh-14* and *fust-1; ceh-14* double mutants to visualize spermatheca morphology. No differences in morphology or development of spermatheca were detected in either double mutant. Representative images show day 1 adults of wild-type and double mutants containing the UX993 transgenes.

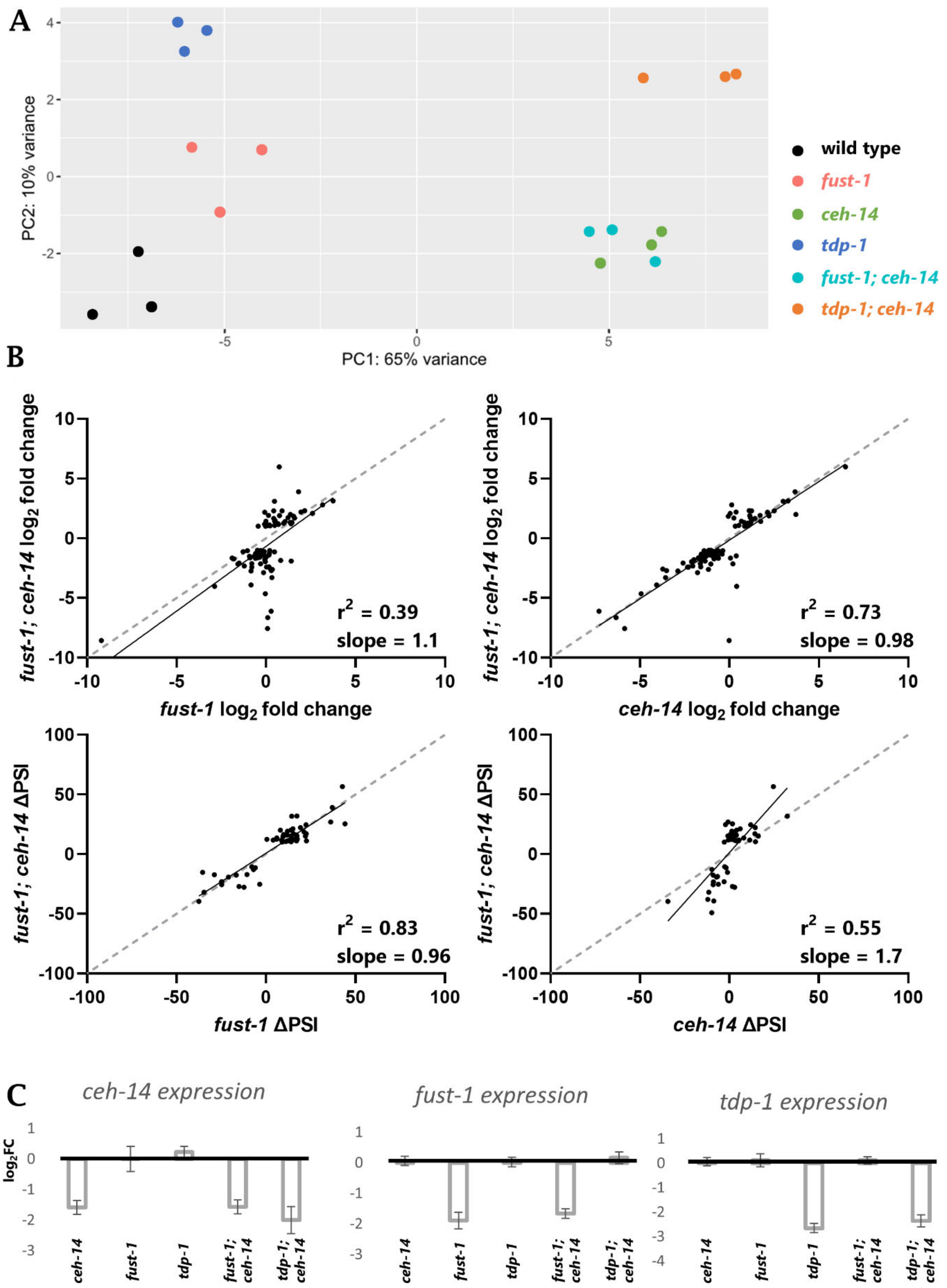

Supplemental Figure 6. *fust-1; ceh-14* exhibit distinct transcriptional and post-transcriptional regulation. (A) PCA plot for each individual RNA Seq replicate (three biological replicates for each genotype) based on gene expression

values using DESeq2 plotPCA. (B) Linear regressions showing gene expression or splicing changes for genes dysregulated (for gene expression|log2fold-change| >2 and q<0.01, and for alternative splicing |ΔPSI|>10, q<.01) in *fust-1; ceh-14* mutants, compared to *ceh-14* and *fust-1* mutants. (C) RNA Seq analysis of mutants shows that *fust-1*, *tdp-1*, and *ceh-14* do not cross-regulate each other at the level of RNA abundance. The only changes in expression occur in the respective mutants (*e.g.* expression of *fust-1* is decreased only in *fust-1* and *fust-1; ceh-14* mutants).

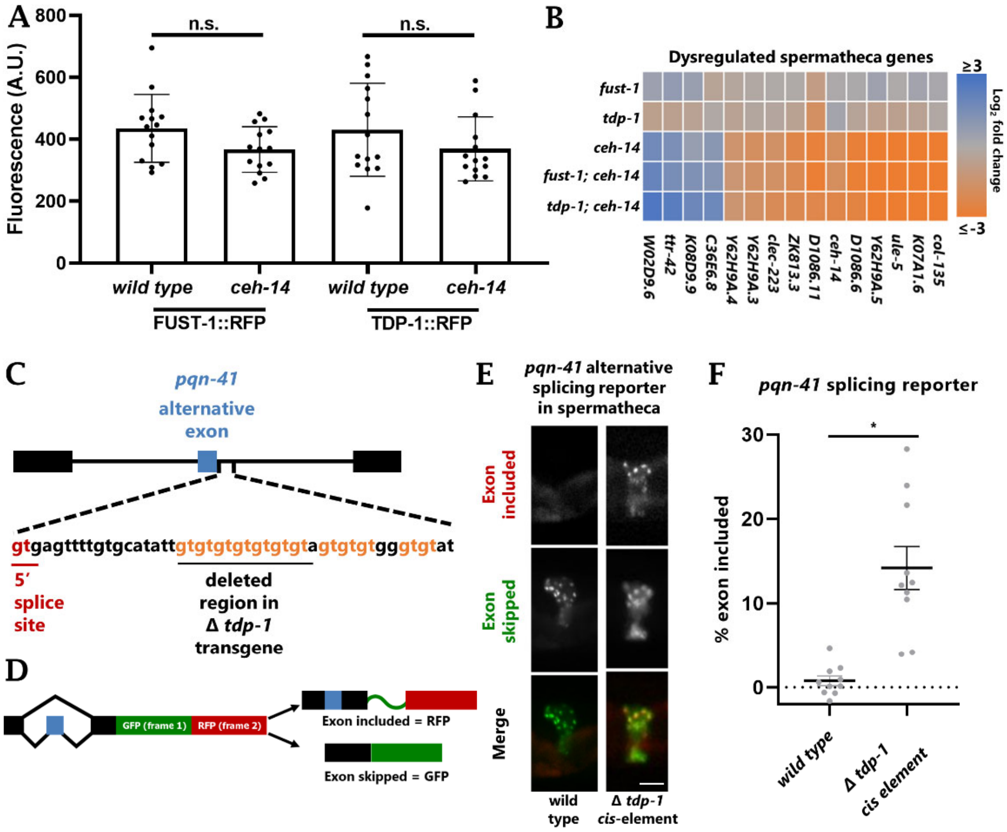

G

Gene	Putative cis element	Location	Δ PSI
STMN2 (human)	tgtgtgagcatgtgtgcgtgtgtg	Exon	+
UNC13A (human)	aggatgtgtggttga and/or atggatgtgtggttgaa	Downstream intron	+
pqn-41	gtgtgtgtgtgtgtagtgtgtggtgtgt	Downstream intron	+ 41%
C06E2.1	gtgtgttgtgcctgt	Upstream intron	+ 37%
F40B5.2	cgtgtgtgcttaacagagtgtga	Upstream intron	+ 26%
W06B4.2	agtctctgtgtgtgaatgcc	Downstream intron	+ 24%
cpna-1	atggtgaccacgtgttgatgtctttggt	Downstream intron	+ 15%

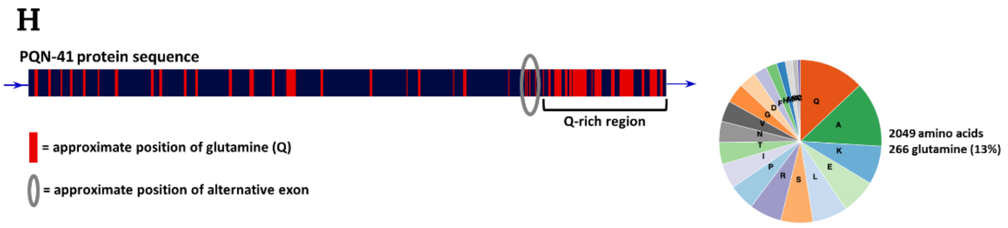

Supplemental Figure 7. Tissue-specific expression of TDP-1/FUST-1 and PQN-41 amino acid sequence. (A) translationally-tagged TDP-1::RFP and FUST-1::RFP show no statistically-significant changes in fluorescence intensity (t-test p > 0.05) in expression in spermathecal nuclei upon loss of *ceh-14*. (B) Similar spermatheca gene dysregulation is seen in *fust-1; ceh-14* and *ceh-14* mutants. As in Fig 5G but with gene names visible. (C) DNA sequence of the intron immediately downstream of the *pqn-41* alternative exon, showing the 7X GT repeat,

followed by 5 additional repeats downstream. The region deleted to generate the *Δ tdp-1 cis* element version of the transgene is underlined. (D) Schematic for the generation of a two-color splicing reporter for *in vivo* tissue-specific visualization of alternative splicing. Exon skipping results in translation of GFP, exon inclusion results in translation of RFP. (E) Representative images of spermatheca for wild type (left) and Δ *tdp-1 cis* element splicing reporter transgenes driven in the spermatheca by the *fkh-6* promoter. Scale bar represents 20 µm. (F) Quantification of spermatheca % exon inclusion according to the formula 100*((RFP intensity)/(RFP intensity + GFP intensity)). $p < 0.05$, t-test. (G) Putative *cis* elements for TDP43/TDP-1-mediated exon inhibition. For reference, two recent human disease-relevant examples are provided, followed by the top TDP-1-regulated cassette exons in worms. In each case, TG-repeat elements are detected in the flanking introns. (H) *pqn-41* amino acid sequence. Approximate locations of glutamine are highlighted in red, and Q-rich region is shown downstream of the circled alternative exon. pqn-41 amino acid composition reveals a bias towards glutamine(97).
